# Closed traumatism of the distal pancreas (A case series of 6 patients)

**DOI:** 10.1016/j.ijscr.2024.110415

**Published:** 2024-10-09

**Authors:** Boubker Idrissi Kaitouni, Youssef Achour, Hamza Ouzzaouit, Omar El Aoufir, Mohammed El Absi, Hamza Sekkat

**Affiliations:** a Digestive Surgical Department, Centre Hospitalier Ibn Sina, Rabat, Morocco; b Faculty of Medicine and Pharmacy, Mohammed V University in Rabat, Morocco; c Central Radiology Department, Centre Hospitalier Ibn Sina, Rabat, Morocco

**Keywords:** Pancreas, Traumatism, Pancreatectomy, Surgery

## Abstract

**Introduction and importance:**

The aim of this retrospective study was to present six cases of trauma to the distal pancreas, highlighting the challenges associated with their diagnosis and management, while underlining their seriousness and the various complications potentially encountered. Our case series highlights individual patient outcomes, demonstrating the diversity of clinical presentations and the importance of customized treatment strategies.

**Case series:**

Between January 2015 and December 2020, six cases of distal pancreas trauma were identified. In two cases, the diagnosis was made based on emergency abdominal CT scans, while in the other four patients, the diagnosis was made directly intraoperatively, mainly because of the severity of the associated lesions, which necessitated laparotomy for exploration.

**Clinical discussion:**

Out of 115 cases of closed abdominal trauma, injury to the distal pancreas was identified in 6 patients, (5.2 %), with a mean age of 21 years. Despite the use of abdominal CT scans for all patients, pancreatic trauma was directly diagnosed intraoperatively in 4 cases (67 %). All patients presented with concomitant abdominal injuries (100 %), and 3 patients (50 %) exhibited multiple severe injuries. Additionally, a significant elevation in pancreatic serum markers was observed in 3 patients (50 %). The pancreatic injuries predominantly involved the tail of the pancreas (67 %), while the body was affected in one patient, and the isthmus was completely transected in another.

Three of our patients developed a pancreatic fistula (50 %) and two patients (33 %) passed away; the first had severe associated lesions, and the second, despite undergoing several iterative laparotomies, succumbed to postoperative complications following a left pancreatectomy.

**Conclusion:**

Closed traumatism of the distal pancreas, although rare, is a significant problem. It is often diagnosed during emergency laparotomy but can sometimes be found on preoperative CT scans. When the patient's condition permits, it is highly advisable to undergo a left pancreatectomy. Simple external drainage is reserved for certain specific situations.

## Highlights

• The aim of this retrospective study was to present six cases of trauma to the distal pancreas, highlighting the challenges associated with their diagnosis and management.
• The management of closed trauma to the distal pancreas is complex and varies greatly from one patient to another. Some authors recommend personalized management based on various parameters.

## 1 Introduction

Closed trauma to the distal pancreas (CTP) is a rare but highly lethal condition. The deep anatomical location of the pancreas within the abdomen, specifically in the retroperitoneal region, makes it challenging to assess due to the surrounding organs [1]. The distal pancreas is enmeshed in a complex vascular and digestive network, leading to a range of potential injuries in this area, including hemorrhage, duodenal involvement, and pure pancreatic contusion [1]. The anatomical complexity of the pancreas and its close associations with neighboring organs-particularly its compact and deep anatomical connection with the spleen contributes to the frequent occurrence of concomitant injuries, such as splenic rupture, which further exacerbate prognosis and increase the risk of complications. These factors necessitate a variety of therapeutic approaches, making the determination of treatment indications a complex task [1]. Additionally, these injuries are often insidious, with a lack of correlation between the severity of the damage and its clinical presentation, leading to delayed diagnosis and further complicating treatment [2].

In recent decades, management of closed pancreatic trauma has evolved considerably thanks to advances in medical imaging, notably the capabilities of computed tomography (CT) or magnetic resonance imaging (MRI) to detect ruptures of the Wirsung duct. To a more limited extent, therapeutic interventions such as endoscopic retrograde Wirsungography, coupled with the application of stents for treating these ruptures, have also played a role in this advancement [1]. However, the decision to undergo laparotomy and the decision to perform pancreatic resection remain of crucial importance in the treatment of these injuries [3,4]. The most appropriate approach is the one that enhances the vital prognosis the most, limits morbidity and hospitalisation time, while preserving the endocrine function of the gland as much as possible. The aim of this study was to report a series of 6 cases and to discuss the diagnostic challenges and therapeutic management of trauma of the distal pancreas.

## 2 Methods

We included all consecutive patients who presented with closed abdominal trauma involving distal pancreatic lesions (i.e., affecting the body, isthmus, or tail of the pancreas) that were diagnosed either radiologically or intraoperatively. The patients were managed within our Visceral Surgical Emergency Department over a five-year period, from January 2015 to December 2020 [Table 1]. In accordance with the PROCESS criteria, for each patient, the following data were collected: age, gender, medical history, circumstances of the trauma, mechanism of injury, site of impact, presence of a free interval, any delays in seeking medical attention or admission, initial symptoms and examination findings upon admission, results from laboratory and radiological investigations (including the American Association for the Surgery of Trauma [AAST] classification), and details from surgical exploration and postoperative monitoring.

**Table 1:** Patient characteristics.

Characteristics	Number (%)
Average age	21 [19–27]
Circumstances of occurrence Road traffic accident Assault with a weapon	4(66 %) 2(33 %)
Clinical presentation Abdominal pain Shock Abdominal contracture	5(83 %) 4(66 %) 2(33 %)
Preoperative CT scan Pancreatic injury Hemoperitoneum Associated injuries	2(33 %) 5(83 %) 5(83 %)
Preoperative lipaemia Positive Negative	3(50 %) 3(50 %)
Emergency surgery	6(100 %)
Distal pancreatic lesion Body and isthmus Tail	2(33 %) 4(66 %)
Associated abdominal injuries Splenic injury Liver injury Kidney injury	6(100 %) 3(50 %) 1(20 %)
Associated extra-abdominal injuries Thoracic injury Cerebral injury	3(50 %) 1(20 %)
Postoperative pancreatitis	3(50 %)
Surgical revision	3(50 %)
Death	2(33 %)

Patients with distal pancreatic lesions associated with open abdominal trauma, as well as those with duodenal-pancreatic trauma, were excluded from the study.

## 3 Case series

Out of 115 cases of closed abdominal trauma, distal pancreas trauma was observed in 6 patients (5.2%).

### 3.1 Case 1

#### 3.1.1 Patient information

A 19-year-old male patient with no significant medical history was involved in a road traffic accident, sustaining an impact to the epigastric region. The patient initially presented at another medical facility 2 h post-accident. At that time, clinical examination findings were normal, and an abdominal CT scan showed no abnormalities.

#### 3.1.2 Clinical presentation

On the third day post-trauma, the patient developed epigastric abdominal pain that rapidly spread to the entire abdomen. Upon admission to our facility, the patient was hemodynamically stable, but physical examination revealed generalized abdominal rigidity.

#### 3.1.3 Diagnostic workup

**Fig. 1:**
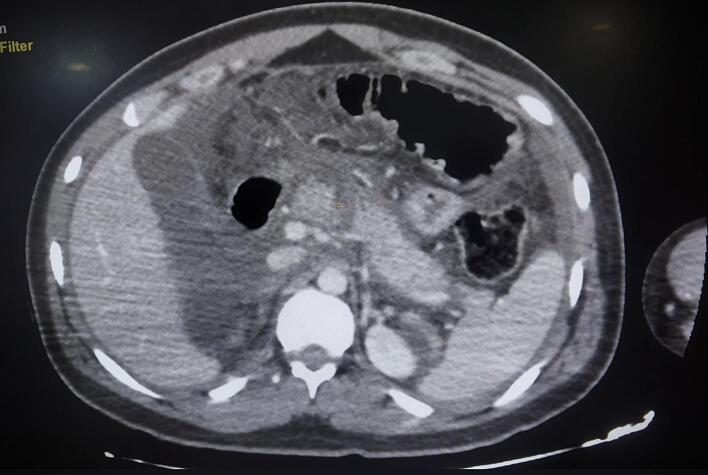
Abdominal CT scan showing complete transection of the isthmus of the pancreas (AAST 3).

Laboratory investigations showed a significant elevation in serum lipase levels, 24 times above the normal range, along with leukocytosis (16,000/mm^3^) and an elevated C-reactive protein (CRP) level of 460 mg/L. Abdominal CT imaging identified a complete parenchymal and ductal transection at the pancreatic isthmus, classified as Lucas grade 3, accompanied by hepatic and splenic injuries classified as grade 2 by the American Association for the Surgery of Trauma (AAST), and a large-volume hemoperitoneum (Fig. 1).

#### 3.1.4 Treatment

**Fig. 2:**
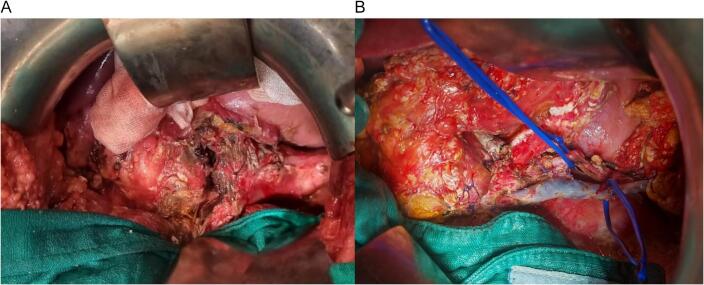
A: Intraoperative photo of isthmic section of the pancreas. B: Photo of a left pancreatectomy with conservation of the spleen (splenic vessels on vascular lakes).

**Fig. 3:**
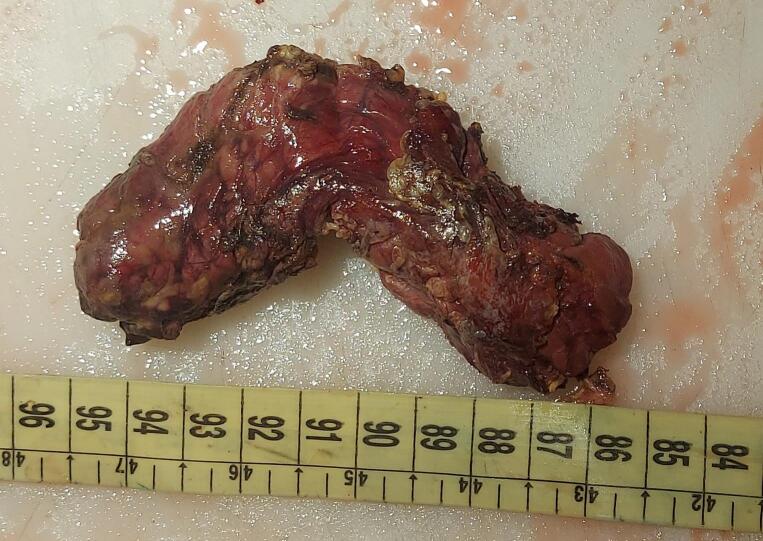
Surgical specimen of a pancreatic resection involving 75 % of the organ.

An emergent laparotomy was decided. Exploration revealed a small amount of peritoneal effusion and complete sectioning of the pancreatic body, with no other associated lesions. A left pancreatectomy was performed with the spleen preserving and suturing the proximal pancreas (Fig. 2, Fig. 3). A large laminar drainage was placed. The immediate postoperative course was uneventful, with the patient spending 72 h in the intensive care unit before being transferred to the general ward.

#### 3.1.5 Outcome

**Fig. 4:**
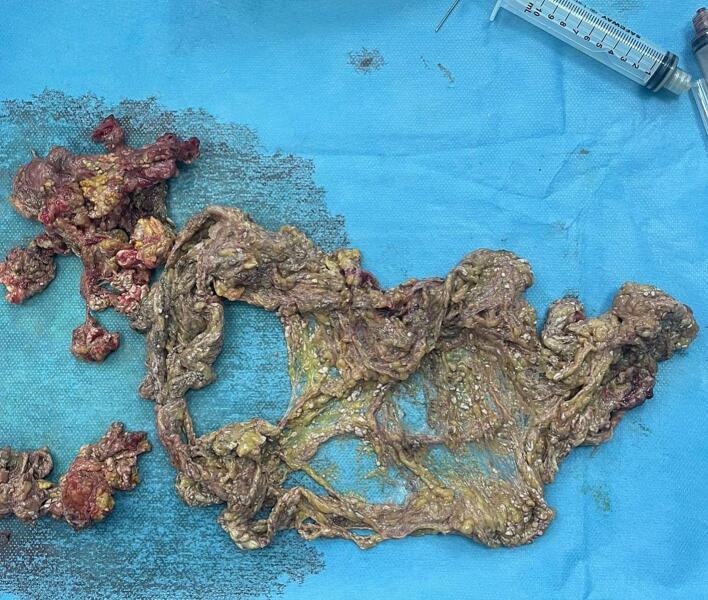
Necrosectomy specimen following postoperative pancreatitis.

Subsequently, the patient developed a pancreatic fistula, indicated by elevated amylase levels in the drain fluid, without affecting the patient's overall condition. Somatostatin therapy was initiated, which resulted in clinical improvement. However, on day 11, the patient developed postoperative pancreatitis, characterized by hemodynamic instability and purulent drainage from the Delbet drains, necessitating a second surgical intervention. Intraoperative findings revealed cytosteatonecrosis of the greater omentum and the pancreatectomy bed. A necrosectomy was performed, along with peritoneal lavage and drainage (Fig. 4).

On day 22, after favorable clinical and laboratory progress, the patient experienced hemorrhagic shock, evidenced by the discharge of hemorrhagic fluid and blood clots from the drains. A third emergency laparotomy was performed, and a hemostatic splenectomy was conducted, along with extensive lavage and drainage.

On day 55, the patient developed a digestive fistula, with substantial contrast extravasation from a defect in the transverse colon in CT scan. As a result, the patient underwent a fourth surgical procedure, during which a transverse colostomy was created following a challenging adhesiolysis. Unfortunately, the patient died 72 h later in the intensive care unit.

### 3.2 Case 2

#### 3.2.1 Patient information

A 19-year-old male with no significant medical history presented as a victim of an assault with a blunt object, sustaining an impact to the left hypochondrium.

#### 3.2.2 Clinical presentation

The patient was admitted to the intensive care unit 4 h post-injury, exhibiting hemodynamic and respiratory instability. Abdominal examination revealed diffuse pain without signs of rigidity.

#### 3.2.3 Diagnostic workup

A CT scan was performed, which demonstrated a left hemothorax and pneumothorax associated with a splenic fracture with an intraparenchymal hematoma and a large hematoma. The pancreas appeared normal on imaging.

#### 3.2.4 Treatment

Due to persistent hemodynamic instability, the patient was taken to the operating room for surgical exploration, which revealed a moderate hemoperitoneum, a laceration of the pancreatic tail, a splenic fracture, a hepatic injury involving the free edge of segments III and IV, and decapsulation of the left kidney. A hemostatic splenectomy was performed, along with a caudal pancreatectomy, closure of the proximal Wirsung duct, and extensive laminar drainage of the abdominal cavity.

#### 3.2.5 Outcome

**Fig. 5:**
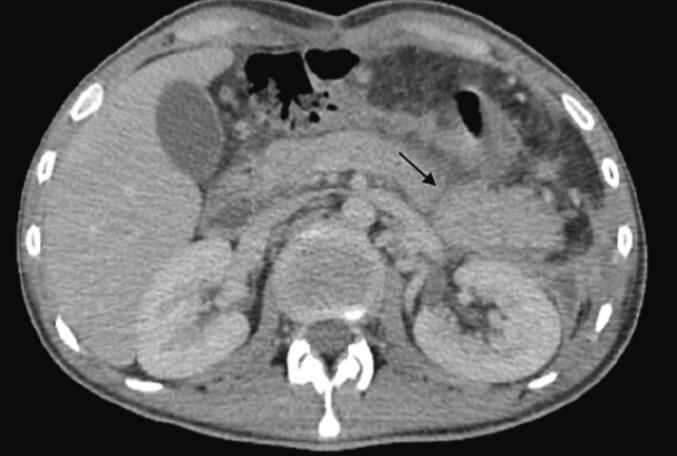
Axial CT section taken on day 10 showing fracture line of the tail of the pancreas.

**Fig. 6:**
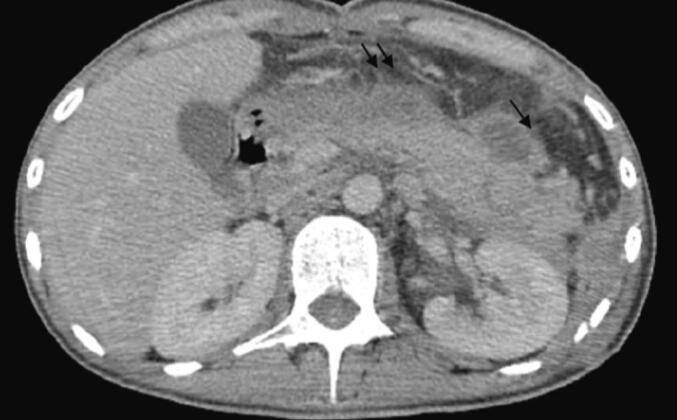
Axial CT section showing acute post-traumatic pancreatitis: peri-pancreatic necrosis and infiltration of peri-pancreatic fat.

On postoperative day 10, the patient developed septic shock, characterized by purulent discharge from the drain and significant inflammatory markers, including leukocytosis (18,000/mm^3^) and elevated C-reactive protein (CRP) levels (370 mg/L), as well as a marked increase in lipase levels from 208 U/L to 898 U/L. An abdominal CT scan was obtained, revealing an abdominal collection sustained by a pancreatic fistula resulting from dehiscence of the remaining stump sutures, as well as post-traumatic hemorrhagic necrotizing pancreatitis (Fig. 5, Fig. 6).

A second surgical intervention was indicated, which involved extensive laminar drainage of the abdominal cavity and around the pancreatic tail. The postoperative course was notable for improvement in both inflammatory and septic conditions.

### 3.3 Case 3

#### 3.3.1 Patient information

A 24-year-old male patient, with no significant medical history, who was involved in a road traffic accident with left thoraco-abdominal and right lower limb impact.

#### 3.3.2 Clinical presentation

The patient was admitted to the emergency room 6 h after the accident. Upon admission, the patient was conscious with a GCS score of 15/15 but was hemodynamically unstable, showing signs of hemorrhagic shock. Abdominal examination revealed diffuse abdominal pain and deformity of the right lower limb without sensory-motor deficits.

#### 3.3.3 Diagnostic workup

After stabilizing the patient, a CT scan was performed, revealing a minimal pneumothorax and a large hemoperitoneum with a splenic fracture and an intra-parenchymal hematoma measuring 6 cm, classified as grade V according to the AAST. The pancreas showed no abnormalities. Additionally, there was a displaced fracture of the right femur.

#### 3.3.4 Treatment

The patient was taken to the operating room, where surgical exploration revealed a large volume hemoperitoneum of 1500 mL, a fractured spleen, and no visible pancreatic abnormalities, justifying hemostatic splenectomy with drain placement and intramedullary nailing of the right femur.

#### 3.3.5 Outcome

**Fig. 7:**
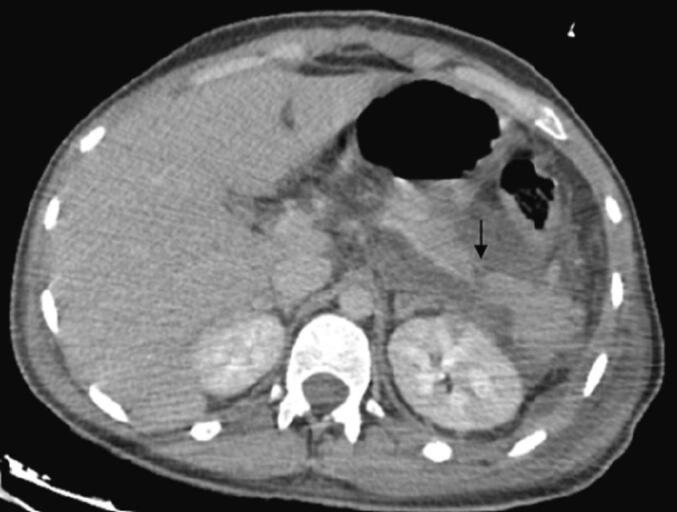
Axial section CT scan on day 3 of the trauma showing a transection of the pancreatic body.

**Fig. 8:**
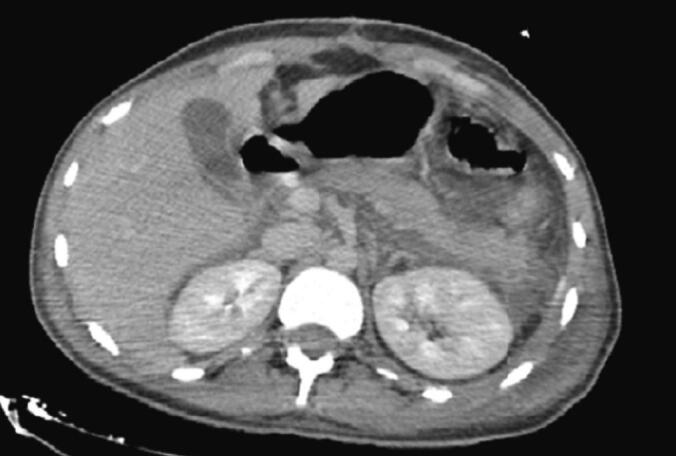
Axial CT section at day 3 showing post-traumatic acute pancreatitis.

In the postoperative period, on day 3, the patient developed a new shock episode and respiratory distress, necessitating transfer to the intensive care unit for intubation and vasoactive drugs. Laboratory tests showed a disrupted biological profile with significant inflammatory and infectious syndromes (leukocytosis = 15,200/mm^3^ and CRP = 250 mg/L). An intra-abdominal postoperative complication was suspected, leading to an abdominal CT scan that revealed swelling of the pancreatic tail with an 8 mm discontinuity in the pancreatic body, classified as grade II according to the AAST, with infiltration of peripancreatic fat and fluid collections in the posterior omental cavity, suggesting a complicated pancreatic fistula with edematous-interstitial pancreatitis (Fig. 7, Fig. 8). This was confirmed biologically by a drain lipase level of 1200 UI/L and a blood lipase level of 215 UI/L.

Exploratory laparotomy could not be performed due to the extreme instability of the patient and the severity of his respiratory condition (ARDS), which prevented transfer to the operating room. The patient's condition deteriorated, and he ultimately passed away.

### 3.4 Case 4

#### 3.4.1 Patient information

This is a 19-year-old male patient with no significant medical history, who was involved in a workplace accident where he was struck and then crushed by a construction machine, with an impact site in the left thoraco-abdominal region.

#### 3.4.2 Clinical presentation

The patient was admitted to the emergency room 8 h after the accident. Upon admission, the patient was conscious with a GCS score of 15/15 but was hemodynamically unstable, showing signs of hemorrhagic shock. Abdominal examination revealed diffuse pain on palpation, particularly pronounced in the left hypochondrium.

#### 3.4.3 Diagnostic workup

**Fig. 9:**
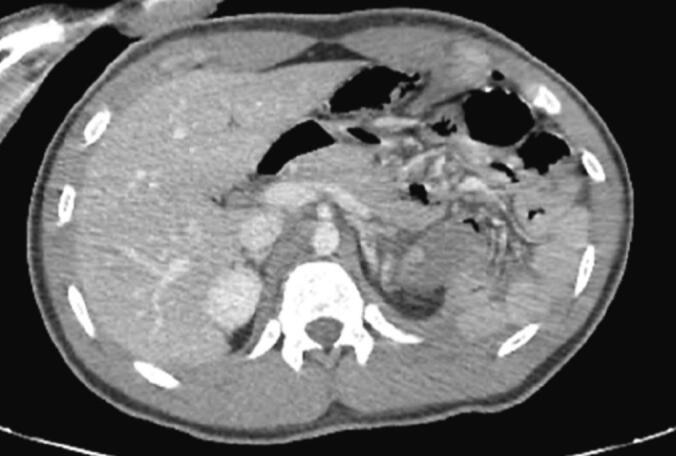
Axial CT section showing a heterogeneous spleen fracture.

After stabilization, a CT scan was performed, revealing a heterogeneous spleen with multiple lacerations, the largest measuring 36 mm, classified as grade III according to the AAST, and no lesions on the pancreas (Fig. 9). The scan also showed a large pneumothorax with pulmonary contusions and rib fractures.

#### 3.4.4 Treatment

The unstable patient received chest tube drainage for the pneumothorax and was then taken urgently to the operating room for exploratory laparotomy. Exploration revealed a small volume hemoperitoneum, a 15 cm breach of the left hemidiaphragm with herniation of the stomach, transverse colon, small intestine, and a fractured and decapsulated spleen, as well as a 1 cm hematoma in the pancreatic tail. The patient underwent a hemostatic splenectomy with placement of a laminar drain in the splenic bed, with preservation of the pancreatic lesion and reduction of the herniated contents, and closure of the diaphragmatic breach.

#### 3.4.5 Outcome

The patient was admitted to the intensive care unit with a favorable postoperative course.

### 3.5 Case 5

#### 3.5.1 Patient information

A 27-year-old male patient with no significant medical history, who was involved in a road traffic accident with an impact site at the left hypochondrium.

#### 3.5.2 Clinical presentation

The patient was admitted to the emergency department 28 h after the accident, presenting with hemorrhagic shock. Initial abdominal examination revealed generalized abdominal rigidity with diffuse abdominal pain with a large bruise over the left hypochondrium, and reflex ileus.

#### 3.5.3 Diagnostic workup

Due to the patient's instability, a bedside abdominal ultrasound was performed, revealing a heterogeneous spleen with a 27 mm deep polar fracture associated with a large hemoperitoneum.

#### 3.5.4 Treatment

An emergency exploratory laparotomy was indicated. Exploration revealed a large-volume hemoperitoneum of 2 l, a spleen fractured at the pedicle, and a small hematoma of the pancreatic tail. The patient underwent a hemostatic splenectomy, with preservation of the caudal pancreatic lesion and thorough lavage of the peritoneal cavity.

#### 3.5.5 Outcome

The postoperative course was uneventful, with good clinical recovery.

### 3.6 Case 6

#### 3.6.1 Patient information

A 18-year-old patient with no significant medical history, was admitted for severe polytrauma involving the cranio-thoraco-abdominal regions following a road traffic accident.

#### 3.6.2 Clinical presentation

The patient was admitted 10 h after the trauma. Upon admission, the examination revealed an unconscious patient with a GCS score of 8, who was stable in terms of respiratory and hemodynamic parameters, with a slightly abnormal abdominal examination.

#### 3.6.3 Diagnostic workup

**Fig. 10:**
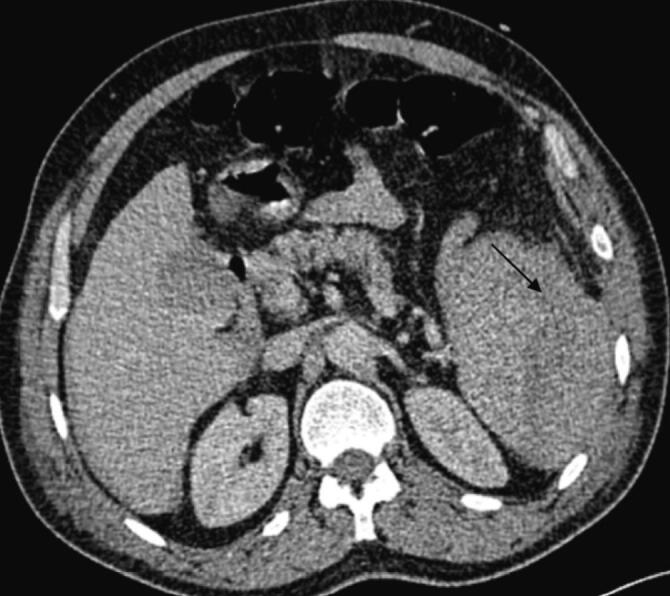
Axial section of the CT scan showing a subcapsular haematoma of the spleen.

**Fig. 11:**
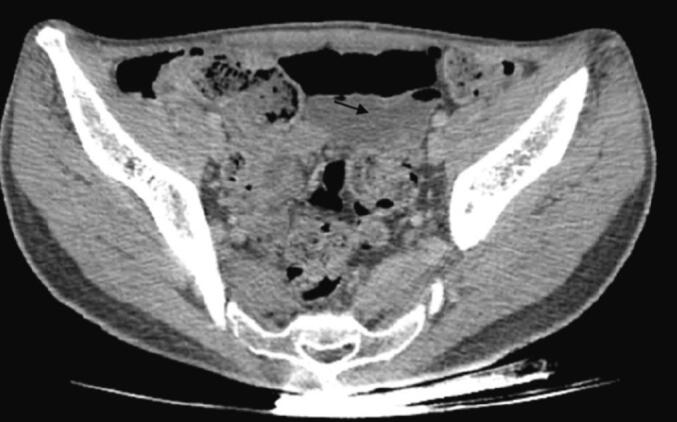
Axial CT section showing pelvic hemoperitoneum.

After rapid stabilization, the patient underwent a whole-body CT. The scan revealed a moderate hemoperitoneum and a fracture of the lower pole of the spleen with involvement of the splenic hilum (Fig. 10, Fig. 11). The pancreas appeared normal on the scan. In addition to the abdominal injuries, multiple cerebral and pulmonary contusion foci were identified, along with radiological evidence of hemothorax.

#### 3.6.4 Treatment

The patient was urgently taken to surgery, where findings included a large hemoperitoneum of 800 cc, splenic contusion with a subcapsular hematoma, liver laceration, and a pancreatic tail contusion with hematoma. A hemostatic splenectomy was performed along with hepatic hemostasis and extensive laminar drainage of the splenic bed, with the pancreatic lesion being preserved.

#### 3.6.5 Outcome

The postoperative course was complicated by evisceration, which required surgical management 20 days after the initial procedure.

## 4 Results

These patients were all men with an average age of 21 years (range: 19–27 years). The primary cause of injury was deceleration shock resulting from road traffic accidents (*n* = 4) or epigastric contusions caused by a knife (*n* = 2). The most common clinical indicators were abdominal tenderness and shock (67 %). Abdominal rigidity was identified in only 2 cases (33 %).

Although all our patients underwent an abdominal CT scan, pancreatic trauma was directly diagnosed intraoperatively in 4 patients (67 %). The diagnosis was established in two patients through abdominal CT scans conducted 3 to 5 days post-trauma. Remarkably, in one of these cases, the initial abdominal CT scan administered just 2 h after the road traffic accident returned entirely normal. However, three days following the traumatic incident, considering the deterioration of the patient's condition, a subsequent CT scan revealed complete transection of the isthmus of the pancreas, along with extensive hemoperitoneum.

All patients exhibited concomitant abdominal lesions involving the liver, spleen, or kidney, while 3 patients (50 %) suffered from multiple severe injuries, including associated cerebral, thoracic, or pulmonary injuries such as rib fractures and hemopneumothorax. Furthermore, a notable elevation in pancreatic serum markers was noted in 3 patients (50 %).

In our series, all patients were urgently admitted to the operating room for an exploratory laparotomy. Pancreatic lesions concerned mainly the tail of the pancreas (67 %), the body was affected in one patient and the isthmus was completely sectioned in another. Ductal rupture of the Wirsung was confirmed in 2 of our patients (33 %), who underwent a left pancreatectomy removing the distal part of the damaged pancreas, with conservation of the spleen. In the other patients, the pancreas was respected, given the grade of pancreatic trauma, and extensive drainage was performed.

The postoperative course was marked by the diagnosis of postoperative pancreatic fistula in 3 patients (50 %), with one of them also experiencing associated postoperative pancreatitis (17 %). Three patients (50 %) necessitated subsequent surgical interventions, 2 of whom underwent iterative surgery for postoperative pancreatitis with lavage and extensive drainage of the abdominal cavity.

Among the 6 patients, one succumbed in intensive care on the 12th day due to associated injuries, while another patient passed away on the 45th day following three subsequent surgeries (the first for postoperative pancreatitis, the second for hemorrhagic shock, and the last for stercoral peritonitis resulting from a perforation of the transverse colon). Consequently, our mortality rate stands at 33 %.

## 5 Discussion

Pancreatic trauma remains uncommon, accounting for around 3–5 % of abdominal trauma in adults [5,6]. A distinct male predominance is evident in numerous studies documented in the literature, primarily impacting young individuals, with approximately 80 % of affected individuals being under the age of 40 [7,8]. Recent data suggest a rise in the incidence of closed traumatic pancreatic injuries (CTP), attributed to the heightened severity of public road accidents and an escalation in civil violence. This increase is fueled by the growing availability of potentially dangerous weapons [9,10]. Closed pancreatic injuries mainly occur during sudden deceleration in drivers who are wearing seat belts or as result of epigastric impact in unbelted drivers hitting the steering wheel additionally to direct epigastric impact with the handlebars in the case of motorcyclists [[10], [11], [12]].

Such traumas can lead to distal pancreatic fractures, where the pancreas impacts the vertebral body, often resulting in damage to the mesenteric vessels on the left side [6]. Due to the violent nature of pancreatic injury and the close proximity of the pancreas to adjacent organs, CTP is seldom an isolated occurrence [6,13,14]. Associated lesions occur in around 50–90 % of cases, with an average of 3.5 organs affected [8,14,15]. These accompanying injuries frequently contribute significantly to the morbidity and mortality associated with CTP. Following closed abdominal trauma, the organs most affected alongside pancreatic injury are the liver, spleen, and duodenum [6].

The morbidity of CTP is closely linked to damage to the Wirsung duct. In practice, serious complications are almost non-existent for simple pancreatic contusion. However, when the pancreatic duct is injured, morbidity can exceed 50 % [16]. The overall mortality rate exhibits significant variability, spanning from 5 % to 30 % across various studies. Direct pancreas damage is accountable for fatalities in only 5–10 % of instances, typically in a delayed fashion, implicating injuries unnoticed during initial evaluations. If diagnosis is delayed, typically occurring between 4- and 8-days post-trauma, the mortality rate can surpass 50 % [[17], [18], [19], [20]].

Hence, a prompt diagnosis is imperative. However, the initial symptomatology's inadequacy and the wide array of clinical presentations often hinder timely diagnosis [21]. Initial clinical manifestations exhibit significant variability, ranging from nearly asymptomatic cases (observed in approximately 20 % of instances in Bradley's study [7]) to pronounced peritoneal manifestations. Typically, clinical examination reveals solely epigastric tenderness [[22], [23], [24]]. Furthermore, symptoms may manifest days or even weeks later [25]. There is no indication suggesting that the initial symptoms reliably signify ductal damage [26]. Certain authors have reported cases of complete pancreas fractures remaining entirely asymptomatic for up to five days post-trauma [25,27].

The lipase and/or amylase test demonstrates limited sensitivity, as evidenced by an elevation in enzyme levels detected at the initial stage in only half of cases [7,28,29]. According to Takishima, this reduced sensitivity may be attributed to the interval between the accident and blood sampling, as hyperlipaemia is observed in 100 % of individuals with pancreatic trauma three hours post-incident [27,30]. Thus, the dynamics of pancreatic enzyme levels prove more informative than the initial absolute value [31].

Abdominal CT scanning remains indispensable for the evaluation of abdominal trauma in hemodynamically stable patients. It stands as the most efficacious complementary examination for establishing a preoperative diagnosis of pancreatic injury [[32], [33], [34], [35]]. Nevertheless, when conducted in the immediate aftermath of the accident, the scan may yield a false negative result in approximately 40 % of cases, as documented by Bradley [7]. If doubts persist about diagnosis despite an initial negative scan, it is crucial to repeat the examination after a few hours. Despite a sensitivity of 85 % within 24 h post-trauma and an overall sensitivity of 90 % [36], CT exhibits limitations in detecting Wirsung duct lesions, with evidence of ductal injury visualized in merely half of cases, often manifesting as a complete parenchymal fracture [37,38].

Several classifications of pancreatic trauma have been proposed by different authors [39,40]. Currently, most specialists adopt the AAST classification (Table 2), focusing solely on pancreatic damage [40] or Lucas's classification, which considers the entire duodenal pancreatic block [41].

**Table 2:** AAST (American Association for the Surgery of Trauma) classification of pancreatic trauma.

Grading	Injury	Description
Grade I	Hematoma Laceration	Mild contusion without duct injury Superficial laceration without duct injury
Grade II	Hematoma Laceration	Major contusion without duct injury Major laceration without duct injury or tissue loss
Grade III	Laceration	Distal transection or parenchymal injury with duct injury
Grade IV	Laceration	Proximal transection or parenchymal injury involving the ampulla
Grade V	Laceration	Massive disruption of the pancreatic head

Magnetic resonance pancreatography (MRP) stands as the preferred method for detecting ductal damage, offering comprehensive visualization of the Wirsung duct. Its efficacy remains moderate during the immediate post-traumatic phase, as Wirsung duct dilatation is not evident even in cases of damage. However, its sensitivity becomes optimal a few days later [42].

Two studies have highlighted the utility of endoscopic retrograde pancreatography (ERP) in assessing the ductal injury presence [43,44]*.* Nonetheless, ERP carries a risk of sepsis and may exacerbate pancreatitis lesions in already compromised tissue [45,46]. Moreover, in 10 % of cases, ERP has failed due to difficulty in catheterizing the papilla, and a recent study reported several false negatives of emergency ERP procedures [45,46]. The primary advantage of ERP lies chiefly in its therapeutic potential, enabling the insertion of a prosthesis into the Wirsung in cases of ductal damage, and the benefit for subsequent investigation and treatment of trauma-related complications [47].

The management of closed trauma to the distal pancreas is complex and varies greatly from one patient to another. Some authors recommend personalized management based on various parameters. [48] A study of 165 patients reported by Laura L and al. provides an overview of the clinical features and outcomes following management of pancreatic trauma. It highlights the importance of appropriate management based on the severity of injury and individual patient characteristics [48]. Harbi Khalayef and al. based on a study of 77 patients, concludes that surgical treatment, although necessary for severe pancreatic trauma, is associated with a higher risk of complications and prolonged recovery. The study recommends an individualized approach based on the severity of injury and patient characteristics to optimize clinical outcomes. [49]

For distal pancreatic injuries, two approaches appear universally accepted. Left pancreatectomy is recommended in cases of complete rupture of the pancreatic duct, necessitating the removal of the pancreas from left to right of the lesion [21]. The decision to proceed with this surgery relies on the patient's overall condition, hemodynamic stability, and local conditions within the abdominal cavity. In cases of AAST grade I pancreatic lesions, simple surveillance may be justified. In cases where these lesions are identified intraoperatively, extensive external drainage of the contusion site might be sufficient [21]. However, this straightforward pancreatic drainage, although sometimes appealing in emergency scenarios, is not advisable in cases of ductal rupture. It can result in severe complications, occasionally requiring subsequent surgery involving delayed and intricate pancreatic resection. These complications are frequently underestimated during the initial surgery, with Lin reporting a morbidity rate of 100 % and a mortality rate of 50 % [42], and Girard's series citing 100 % morbidity and 20 % mortality [31]. Most authors recommend distal pancreatic resection, as it yields significantly lower mortality rates, morbidity, and hospital stay durations compared to external drainage of pancreatic rupture [19,42,50].

Left splenopancreatectomy, extended to varying degrees towards the right, emerges as is the most suitable procedure in emergency situations due to its simplicity and swiftness. However, sacrificing the spleen, often done for operational convenience, can lead to significant immunological consequences in the future [51]. When feasible, preserving the spleen may be advisable, especially in young trauma patients [[52], [53], [54], [55]]. Although the option of anastomosing the pancreatic stump to a jejunal loop has been proposed, it lacks demonstrated benefits and is an inappropriate and time-consuming procedure, particularly in patients with multiple traumas [[55], [56], [57]].

When the pancreatic lesion has not been identified initially or when non-surgical alternatives are unsuitable, pancreatic contusion may progress to acute post-traumatic infected pancreatitis, characterized by the formation of deep abscesses or the onset of post-traumatic peritonitis [7,58]. This form of pancreatitis poses a significant threat to life, with a mortality rate reaching up to 40 % [17,20]. In instances of severe pancreatitis, exploratory laparotomy becomes imperative for lesion characterization, potential necrosectomy, and placement of necessary drains. Crucially, this exploration serves to rule out any suspicion of other associated lesions, particularly within the digestive tract. Post-operative morbidity is mainly related to the risk of developing an external pancreatic fistula, with an estimated incidence ranging between 10 % and 20 % [6,59,60]. It is noteworthy that spontaneous fistula healing probability is less than 20 % [11,61].

While these pancreatic fistulas may persist, they typically subside almost systematically within four months [59]. Their medical management parallels that of postoperative pancreatic fistulas following pancreatic resection. Even though the preventive use of somatostatin in this traumatic context has been subject to investigation in small trials yielding conflicting results, its usage may be justified. Its primary aim is to reduce the output of pancreatic fistulas. [5,63].

This study differs from existing research in that it provides a detailed analysis of six cases of blunt pancreatic trauma, offering a comprehensive view of the diagnostic challenges, surgical management, and postoperative care strategies specific to this rare but critical injury. Unlike broader studies that may generalize findings, our case series focuses on individual patient outcomes, highlighting the variability in clinical presentation and the tailored approaches required. The main limitations of our study come from the small size of our cohort and its monocentric nature making the external validity of our data reduced. The retrospective collection of data from patients' medical records is another limitation, as the accuracy of the information depends heavily on the precision of these records. An extension of this investigation is planned to further analyze the morbidity and mortality associated with each surgical procedure.

## 6 Conclusion

Although mortality related to pancreatic trauma often stems from associated injuries, delayed diagnosis of severe pancreatic injury significantly worsens prognosis. Emergency laparotomy should consistently adhere to the “damage control” principle. The status of the Wirsung duct damage crucially influences treatment selection and prognosis for closed trauma to the distal pancreas. In cases of duct damage, prompt left pancreatectomy should be executed, contingent upon the patient's overall condition. In instances of hemodynamic instability, temporary external drainage may be employed while awaiting more extensive secondary surgery. A non-surgical approach to a confirmed rupture of the Wirsung duct is feasible only in stable clinical conditions. This approach necessitates ongoing monitoring, optimal utilization of resources provided by CT and/or MRI scans, and close collaboration with interventional endoscopy, particularly in specialized institutions.

## Consent

Written informed consent was obtained from the patients for publication of this case report and accompanying images. A copy of the written consent is available for review by the Editor-in-Chief of this journal on request.

## Ethical approval

Ethical approval for this study was provided by the Ethical Committee of Ibn Sina University Hospitals, Mohammed V University, Rabat, Morocco on 06 March 2024.

## Funding

The authors have no source of funding or financial support except themselves.

## Author contribution

HS designed the paper. BIK and YA collected the data, BIK, YA and HO wrote the first draft of the manuscript.

HS participated in the article design and critically reviewed the manuscript. SB, MEA, and HS critically reviewed the manuscript. All authors approved the final version of the manuscript.

BIK and YA made the changes and introduced the detailed study.

HS and SB supervised and corrected the changes.

## Guarantor

Boubker Idrissi Kaitouni.

## Research registration number

https://orcid.org/0009-0005-4280-4204 **IJSCR_110415**

## Conflict of interest statement

The authors have no conflicts of interest and source of funding. The subject of study had no commercial interest, no financial or material support.

## Data Availability

The data that support the findings of patient information are available from the corresponding author upon reasonable request.

## References

[1] Girard E., Abba J., Letoublon C., Arvieux C. (2017). Traumatismes du pancréas et du duodénum – Principes de prise en charge et techniques chirurgicales. EMC-Techniques chirurgicales - Appareil digestif.

[2] Pradère B., Carrère N., Gouzi J.L. (Déc 1999). Techniques chirurgicales. Les pancréatectomies gauches. J. Chir. (Paris).

[3] Barkin J.S., Ferstenberg R.M., Panullo W., Manten H.D., Davis R.C. (1988). Endoscopic retrograde cholangiopancreatography in pancreatic trauma. Gastrointest. Endosc..

[4] Kim H.S., Lee D.K., Kim I.W., Baik S.K., Kwon S.O., Park J.W. (2001). The role of endoscopic retrograde pancreatography in the treatment of traumatic pancreatic duct injury. Gastrointest. Endosc..

[5] Pata G., Casella C., Di Betta E., Grazioli L., Salerni B. (2009). Extension of nonoperative management of blunt pancreatic trauma to include grade III injuries: a safety analysis. World J. Surg..

[6] Krige J.E.J., Beningfield S.J., Nicol A.J., Navsaria P. (2005). The management of complex pancreatic injuries. S. Afr. J. Surg..

[7] Bradley E.L., Young P.R., Chang M.C., Allen J.E., Baker C.C., Meredith W. (1998). Diagnosis and initial management of blunt pancreatic trauma: guidelines from a multi-institutional review. Ann. Surg..

[8] Feliciano D.V., Martin T.D., Cruse P.A., Graham J.M., Burch J.M., Mattox K.L. (1987). Management of combined pancreatoduodenal injuries. Ann. Surg..

[9] Chrysos E., Athanasakis E., Xynos E. (2002). Pancreatic trauma in the adult: current knowledge in diagnosis and management. Pancreatology.

[10] Wilson R.H., Moorehead R.J. (1991). Current management of trauma to the pancreas. Br. J. Surg..

[11] Jobst M.A., Canty T.G., Lynch F.P. (1999). Management of pancreatic injury in pediatric blunt abdominal trauma. J. Pediatr. Surg..

[12] Glancy K.E. (1989). Review of pancreatic trauma. West. J. Med..

[13] Vasquez J.C., Coimbra R., Hoyt D.B., Fortlage D. (2001). Management of penetrating pancreatic trauma: an 11-year experience of a level-1 trauma center. Injury.

[14] Akhrass R., Yaffe M.B., Brandt C.P., Reigle M., Fallon W.F., Malangoni M.A. (1997). Pancreatic trauma: a ten-year multi-institutional experience. Am. Surg..

[15] Stone H.H., Fabian T.C., Satiani B., Turkleson M.L. (1981). Experiences in the management of pancreatic trauma. J. Trauma.

[16] Krige J.E., Kotze U.K., Setshedi M., Nicol A.J., Navsaria P.H. (2015). Prognostic factors, morbidity and mortality in pancreatic trauma: a critical appraisal of 432 consecutive patients treated at a level 1 trauma Centre. Injury.

[17] Johanet H., Fasano J.J., Marmuse J.P., Fichelle A., Saint-Marc O., Benhamou G. (1991). Pancreatic trauma: diagnostic and therapeutic emergency. Apropos of 35 cases. J. Chir..

[18] Farrell R.J., Krige J.E., Bornman P.C., Knottenbelt J.D. (1996). Operative strategies in pancreatic trauma. Br. J. Surg..

[19] Patton J.H., Lyden S.P., Croce M.A., Pritchard F.E., Minard G., Kudsk K.A. (1997). Pancreatic trauma: a simplified management guideline. J. Trauma.

[20] Errougani A., Ameur A., Chkoff R., El Alj A., Balafrej S. (1997). Duodenopancreatic injuries. A propos of 30 cases. J. Chir..

[21] Zerbib P., Brams A., Chambon J.P. (2001). Les fractures isthmiques du pancréas. Ann. Chir..

[22] Kielen J., de la Coussaye J.E. (1999). Prise en charge d’un polytraumatisé. J. Chir..

[23] Wilson R.H., Moorehead R.J. (1991). Current managment of trauma. Br. J. Surg..

[24] Jurczak F., Kahn X., Letessier E., Plattner V., Héloury Y., Le Néel J.C. (1999). Traumatismes fermés duodénopancréatiques sévères. À propos d’une série de 30 patients. Ann. Chir..

[25] Boudet M.J. (1998). Traumatisme du pancréas à huit clos. Gastro Méd. Staff.

[26] Nadler E.P., Gardner M., Schall L.C., Lynch J.M., Ford H.R. (1999). Management of blunt pancreatic injury in children. J. Trauma Acute Care Surg..

[27] Taskishima T., Sugimoto K., Hirata M., Asari Y., Ohwada T., Kakita A. (1997). Serum amylase level on admission in the diagnosis of blunt injury to the pancreas. Ann. Surg..

[28] Carrel T., Lerut J., Niederhauser U., Schweizer W., Blumgart L.H. (1990). Diagnosis and treatment of traumatic injuries of the duodenum and pancreas: 21 cases. J. Chir..

[29] Bouwman D.L., Weaver D.W., Walt A.J. (1984). Serum amylase and its isoenzymes: a clarification of their implications in trauma. J. Trauma.

[30] Vitale G.C., Larson G.M., Davidson P.R., Bouwman D.L., Weaver D.W. (1987). Analysis of hyperamylasemia in patients with severe head injury. J. Surg. Res..

[31] Girard E., Abba J., Arvieux C., Trilling B., Sage P.Y. (2016). Management of pancreatic trauma. J. Visc. Surg..

[32] Chambon J.P., Quandalle P., Lemaitre L., Wurtz A., Sobecki L., Saudemont A. (1990). La tomodensitométrie de l’abdomen dans huit cas de traumatisme pancréatique. Ann. Chir..

[33] Patton J.H., Fabian T.C. (1996). Complex pancreatic injuries. Surg. Clin. North Am..

[34] Marsot-Dupuch K., Fery E., Frileux P., Muntlak H., Wind P., Tubiana J.M. (1988). Tomodensitométrie et traumatismes pancréatiques. À propos de 17 cas. Ann. Radiol..

[35] Procacci C., Graziani R., Bicego E., Mainardi P., Bergamo. (1997). Blunt pancreatic trauma. Role of CT. Acta Radiol..

[36] Patel S.V., Spencer J.A., El-Hasani S., Sheridan M.B. (1998). Imaging of pancreatic trauma. Br. J. Radiol..

[37] Gupta V., Wig J.D., Garg H. (2008). Trauma pancreaticoduodenectomy for complex pancreaticoduodenal injury. Delayed reconstruction. J. Pancreas.

[38] Weishaupt D., Grozaj A.M., Willmann J.K., Roos J.E., Hilfiker P.R., MarincekB. (2002). Traumatic injuries: imaging of abdominal and pelvic injuries. Eur. Radiol..

[39] Perissat J., Collet D., Arnoux R., Salloum J., Bikandou G. (1991).

[40] Moore E.E., Cogbill T.H., Malangoni M.A., Jurkovich G.J., Champion H., Gennarelli T.A. (1990). Organ injury scaling 2: pancreas, duodenum, small bowel, colon, rectum. J. Trauma.

[41] Lucas C.E. (1977). Diagnosis and treatment of pancreatic and duodenal injury. Surg. Clin. North Am..

[42] Lin B.C., Chen R.J., Fang J.F., Hsu Y.P., Kao J.L. (2004). Management of blunt major pancreatic injury. J. Trauma.

[43] Taxier M., Sivak M.V., Cooperman A.M. (1980). Endoscopic retrograde pancreatography in the evaluation of trauma to the pancreas. Surg. Gynecol. Obstet..

[44] Whittwell A.E., Gomez G.A., Byers P. (1989). Blunt pancreatic trauma: prospective evaluation of early endoscopic retrograde pancreatography. South. Med. J..

[45] Nirula R., Velmahos G.C., Demetriades D. (1999). Magnetic resonance cholangiopancreatography in pancreatic trauma: a new diagnostic modality?. J. Trauma.

[46] Wind P., Tiret E., Cunningham C., Frileux P., Cugnenc P.H., Parc R. (1999). Contribution of endoscopic retrograde pancreatography in management of complications following distal pancreatic trauma. Am. Surg..

[47] Wind P., Chevallier J.M., Sauvanet A., Delmas V., Cugnenc P.H. (1996). Anatomic basis of mesenteric elongation for ileo-anal anastomosis with J-shaped reservoir: comparison of two techniques of vascular section. Surg. Radiol. Anat..

[48] Meijer Laura L. (2023 Jun). Clinical characteristics and long-term outcomes following pancreatic injury – an international multicenter cohort study. Heliyon.

[49] Khalayleh Harbi (May 31 2022). An analysis of 77 cases of pancreatic injuries at a level one trauma center: outcomes of conservative and surgical treatments. Ann. Hepatobiliary Pancreat. Surg..

[50] Krige J.E.J., Kotze U.K., Hameed M., Nicol A.J., Navsaria P.H. (2011). Pancreatic injuries after blunt abdominal trauma: an analysis of 110 patients treated at a level 1 trauma centre. S. Afr. J. Surg..

[51] Arvieux C., Reche F., Breil P., Létoublon C. (2009).

[52] Meier D.E., Coln C.D., Hicks B.A., Guzzetta P.C. (2001). Early operation in children with pancreas transection. J. Pediatr. Surg..

[53] Wood J.H., Partrick D.A., Bruny J.L., Sauaia A., Moulton S.L. (2010). Operative vs nonoperative management of blunt pancreatic trauma in children. J. Pediatr. Surg..

[54] McGahren E.D., Magnuson D., Schaller R.T. (1995). Management of transected pancreas in children. Aust. N. Z. J. Surg..

[55] Ruszinkó V., Willner P., Oláh A. (2005). Pancreatic injury from blunt abdominal trauma in childhood. Acta Chir. Belg..

[56] Chinnery G.E., Thomson S.R., Ghimenton F., Anderson F. (2008). Pancreatico-enterostomy for isolated main pancreatic duct disruption. Injury.

[57] Hamidian Jahromi A., D’Agostino H.R., Zibari G.B., Chu Q.D., Clark C., Shokouh-Amiri H. (2013). Surgical versus nonsurgical management of traumatic major pancreatic duct transection: institutional experience and review of the literature. Pancreas.

[58] Stringer M.D. (2005). Pancreatitis and pancreatic trauma. Semin. Pediatr. Surg..

[59] Sharpe J.P., Magnotti L.J., Weinberg J.A., Zarzaur B.L., Stickley S.M., Scott S.E. (2012). Impact of a defined management algorithm on outcome after traumatic pancreatic injury. J. Trauma Acute Care Surg..

[60] Lin B.C., Liu N.J., Fang J.F., Kao Y.C. (2006). Long-term results of endoscopic stent in the management of blunt major pancreatic duct injury. Surg. Endosc..

[61] Carr N.D., Cairns S.J., Lees W.R., Russell R.C. (1989). Late complications of pancreatic trauma. Br. J. Surg..

[63] Nwariaku F.E., Terracina A., Mileski W.J., Minei J.P., Carrico C.J. (1995). Is octreotide beneficial following pancreatic injury?. Am. J. Surg..

